# Biological potential of thiazolidinedione derivatives of synthetic origin

**DOI:** 10.1186/s13065-017-0357-2

**Published:** 2017-12-08

**Authors:** Sucheta, Sumit Tahlan, Prabhakar Kumar Verma

**Affiliations:** Department of Pharmaceutical Sciences, Faculty of Pharmaceutical Sciences, Maharshi Dayanand University, Rohtak, Haryana 124001 India

**Keywords:** Thiazolidinedione derivatives, Antidiabetic, Antimicrobial, Anti-inflammatory

## Abstract

Thiazolidinediones are sulfur containing pentacyclic compounds that are widely found throughout nature in various forms. Thiazolidinedione nucleus is present in numerous biological compounds, e.g., anti-malarial, antimicrobial, anti-mycobacterium, anticonvulsant, antiviral, anticancer, anti-inflammatory, antioxidant, anti-HIV (human immunodeficiency virus) and antitubercular agent. However, owing to the swift development of new molecules containing this nucleus, many research reports have been generated in a brief span of time. Therefore seems to be a requirement to collect recent information in order to understand the current status of the thiazolidinedione nucleus in medicinal chemistry research, focusing in particular on the numerous attempts to synthesize and investigate new structural prototypes with more effective antidiabetic, antimicrobial, antioxidant, anti-inflammatory, anticancer and antitubercular activity.
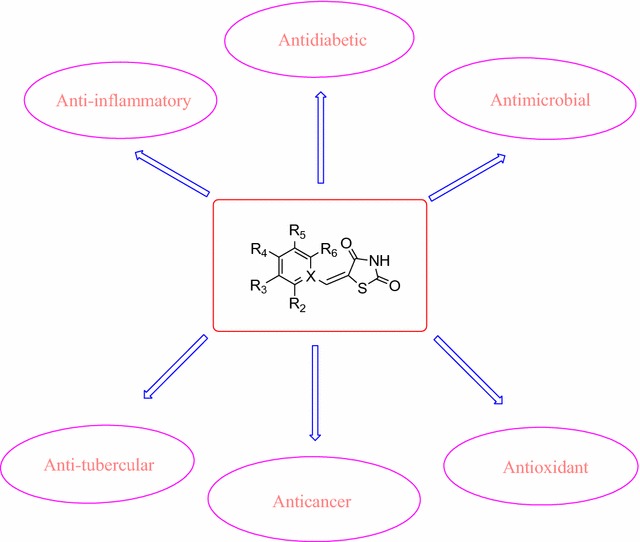

## Introduction

The number of antimicrobial drugs available in the market is vast, but there is a need to discover novel antimicrobial agents with better pharmacodynamic and pharmacokinetic properties with lesser or no side effects. Most of thiazolidinediones exhibit good bactericidal activity against various Gram-positive and Gram-negative bacteria. The bactericidal activity of thiazolidinediones derivatives depends on the substitution on the heterocyclic thiazolidine ring rather than the aromatic moiety.

**Scheme 1:**
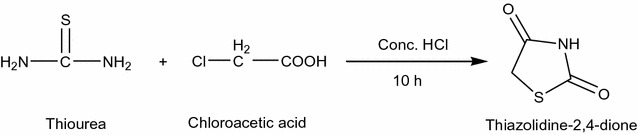
Synthesis of Substituted thiazolidine-2,4-dione

Thiazolidinedione (Scheme 1) along with their derivatives draw attention as they have diverse biological as well as clinical use. Researchers focus on this moiety because it is involved in the control of various physiological activities. Heterocyclic moieties having Nitrogen and Sulfur are involved in a broad range of pharmacological processes. This created interest among researchers who have synthesized variety of thiazolidinediones derivatives and screened them for their various biological activities. In the present study, we have made an attempt to collect biological properties of thiazolidinediones and its derivatives of synthetic origin.

## Biological activities of thiazolidinediones derivatives in the new millennium

### Thiazolidinedione derivatives as antidiabetic agents

Diabetes mellitus (DM), also known as diabetes, is represented by the high blood sugar level over a period of prolonged time. There are three types of diabetes: (i) type 1 DM in which pancreas fails to produce insulin. Previously, it was referred as “insulin-dependent diabetes mellitus” or “juvenile diabetes”, (ii) type-2 DM a condition in which cells does not respond to insulin. Previously, it was referred as “non insulin-dependent diabetes mellitus”, (iii) gestational diabetes is the third main type and arises in pregnant women with no prior record of diabetes with high blood sugar levels [1].

The fundamental reasons of diabetes are a low production of insulin, the inability of the body to use it, or a combination of both (hormone which regulate carbohydrate, fat and protein metabolism). Normally it is a long-standing syndrome having different clinical revelation, with a number of problems such as cardiovascular, hypertension, renal, neurological. It is a disease in which pancreas does not secrete sufficient insulin or cells prevent reacting toward secreted insulin, that’s why cells cannot absorb blood glucose. Its symptoms are recurrent urination, tiredness, too much dehydration and hunger. It is cured by change in food habits, by regulation of proper diet; oral prescription and few situations include insulin injection [2, 3]. The thiazole moiety is a significant heterocyclic unit in drug invention. Literature survey shows that the wide-spread studies have been carried out on the production of thiazolidinediones. Thiazolidiones compounds shows a number of pharmacological activities such as antimicrobial, antitubercular, anti-tumor, anti-viral, anti-HIV, anti-inflammatory and anti-diabetic effects [4–6].

**Scheme 2:**
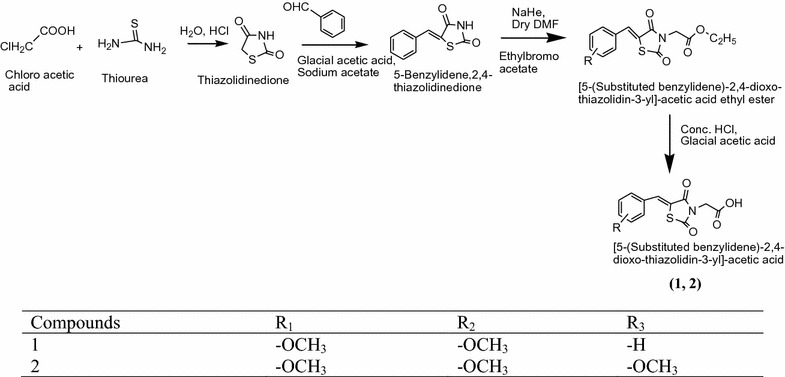
Synthesis of [5-(Substituted benzylidene)-2,4-dioxo-thiazolidin-3-yl]-acetic acid

Datar et al. [7] synthesized a new series of thiazolidinediones by the reaction of thiazolidenedione with several benzaldehyde derivatives using Scheme 2. In vitro anti-diabetic activity of synthesized compound was performed by SLM model. In this series compounds **1** and **2** found to be most active [5-(3,4-dimethoxy)benzylidine-2,4-thiazolidinedione,5-(3,4,5 trimethoxy)benzylidine-2,4-thiazolidenedione] due to presence of methoxy group and comparable to standard drug pioglitazone studies. The results of the most active compound are indicated Tables 1 and 2 (Datar et al. [7]).

**Table 1:** Blood glucose level in experimental animals (mg/dl)

Compounds	Time (min)				
	0	30	60	90	120
DMSO	145	150	150	147	141
Pioglitazone	139	105	110	112	115
**1**	141	112	117	118	112
**2**	147	110	112	107	104

**Table 2:** Decrease in blood glucose levels by AUC method

Compounds	Time (min)				
	30	60	90	120	% reduction in blood glucose level
DMSO	+ 11	+ 05	+ 02	− 04	+ 31
Pioglitazone	− 34	− 39	− 29	− 26	− 23.07
**1**	− 29	− 25	− 24	− 27	− 21.71
**2**	− 37	− 35	− 28	− 24	− 22.84

**Scheme 3:**
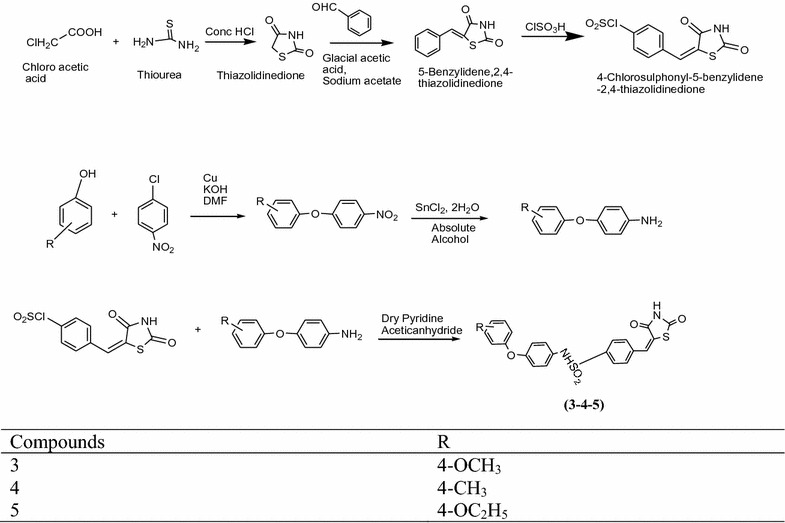
Synthesis of 5-[4-Substituted) sulphonyl benzylidene]-2,4-thiazolidinedione

Swapna et al. [8] synthesized novel thiazolidinediones by using Scheme 3. In vitro antidiabetic activity performed by alloxan induced tail tipping method. From this series compound **3**, **4**, **5** showed highest activity as comparable to standard drug metformin because of presence of electron donating group. The results of most active derivatives showed in Table 3 (Swapna et al. [8]).

**Table 3:** Blood glucose level (mg/dl) of synthesized thiazolidinediones derivatives

Compounds	Blood glucose level (mean ± SE)		
	0 h	3 h	6 h
**3**	343 ± 5.797	313.8 ± 9.411	303.2 ± 9.827
**4**	341.5 ± 6.158	320.5 ± 6.737	313 ± 9.500
**5**	353.7 ± 6.026	315.8 ± 8.109	311.2 ± 9.297
Positive control	335.7 ± 5.168	345.5 ± 5.488	354 ± 8.135
Normal control	125.0 ± 4.497	126.3 ± 4.047	127.7 ± 3.703
Metformin	343.3 ± 6.206	322.8 ± 4.989	292.0 ± 7.767

**Scheme 4:**
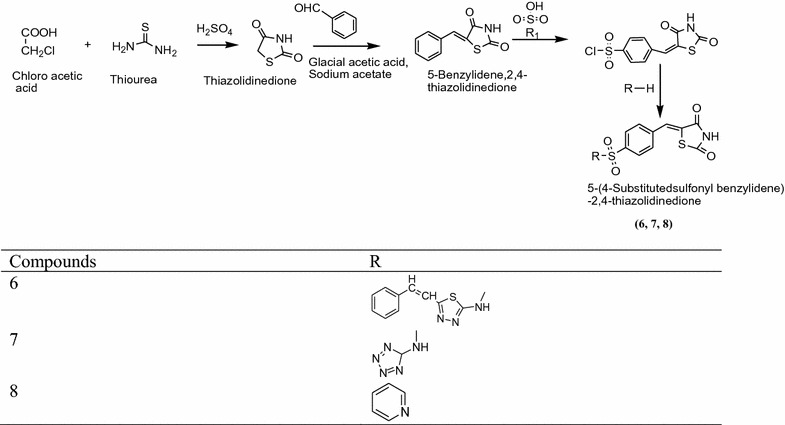
Synthesis of 5-(4-Substituted sulfonyl benzylidene)-2,4-thiazolidenedione

Pattan et al. [2] synthesized a new series of thiazolidinediones derivatives [5-(4-substitutedsulfonylbenzylidene)-2,4-thiazolidinedione] using Scheme 4. The In vitro antidiabetic activity performed by ANOVA and Dunnet’s ‘t’ test. From this series **6**, **7** and **8** compound showed moderates activity and comparable to the standard drug glibenclamide. The results of active compound are given in Table 4 (Pattan et al. [2]).

**Table 4:** Blood glucose level (mg/dl) in synthesized compounds

Compounds	Blood glucose level (mean ± SE)			
	0 h	1 h	3 h	6 h
**6**	320.5 ± 15.81	145.5 ± 2.26	137.0 ± 3.80	123.5 ± 1.10
**7**	213.5 ± 8.78	140.7 ± 3.30	106.3 ± 6.91	95.75 ± 6.06
**8**	283.5 ± 43.76	205.75 ± 49.7	166.3 ± 38.92	124.5 ± 13.16
Standard	385.8 ± 21.37	230.8 ± 12.35	156.8 ± 10.87	93.4 ± 4.98

**Scheme 5:**
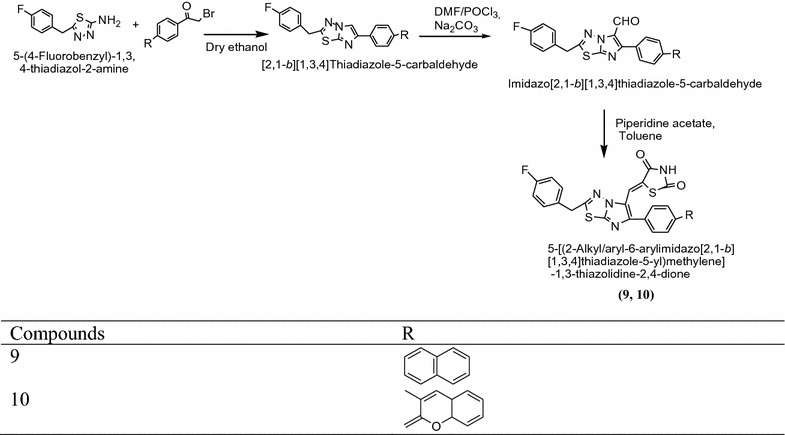
Synthesis of 5-{[2-(4-Fluorobenzyl)-6-arylimidazo[2,1-*b*] [1, 3, 4] thiadiazol-5-yl]methylene}thiazolidine-2,4-diones

Badiger et al. [9] synthesized novel thiazolidinediones derived from 4-fluorophenylacetic acid and thiosemicarbazide in phosphorous oxychloride using Scheme 5. The in vitro antidiabetic activity of synthesized compound [5-{[2-(4-alkyl/aryl)-6-arylimidazo[1,2][1,3,4]thiadiazol-5-yl]metylene}-1,3-thiazolidine-2,4-dione] were performed by alloxan induced tail tipping method. Among them, compounds **9** and **10** found to be most active due to presence of napthyl and coumarinyl groups at C_5_ position as compared to standard drug pioglitazone. The results of synthesized compounds presented in Table 5 (Badiger et al. [9]).

**Table 5:** Plasma glucose level of 3–4 at various drug doses

Compounds	% decrease in plasma glucose level (PG) at various drug doses (mg/kg bodyweight)		
	10 mg	30 mg	60 mg
**9**	42.48 + 3.25	62.24 + 3.42	70.35 + 3.14
**10**	45.42 + 1.25	58.36 + 2.36	68.42 + 2.16
Pioglitazone	47.25 + 5.50	64.59 + 5.42	75.43 + 3.40

**Scheme 6:**
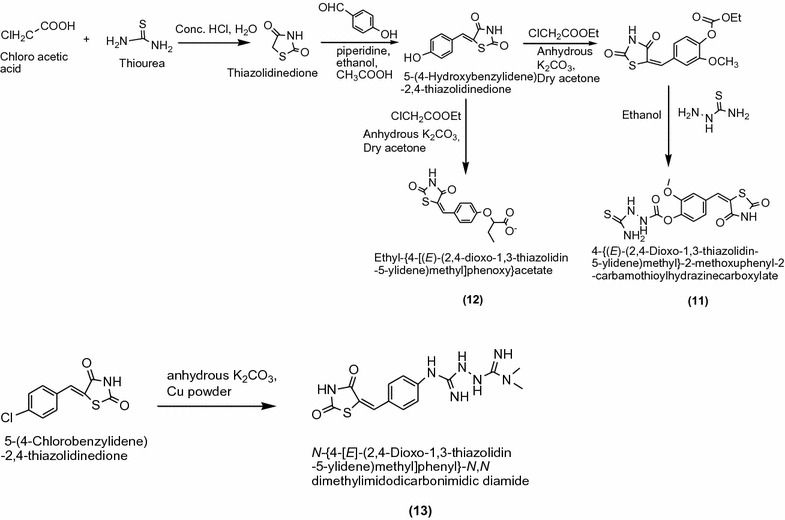
Synthesis of 5-(Substituted benzylidene)-2,4-thiazolidinedione

Patil et al. [10] synthesized a new series of thiazolidinedione derivatives derived from thiourea and chloroacetic acid in ethanol/DMF as presented in Scheme 6. The In vitro antidiabetic activity of synthesized compounds was performed by alloxan induced tail tipping method. From these series compounds **11**, **12** and **13** showed better activities compared to pioglitazone and metformin as standard drug. The results of most active derivatives showed in Table 6 (Patil et al. [10]).

**Table 6:** Hypoglycemic effect of synthesized compounds

Compounds	Blood glucose level mg/dl (mean ± SE)			
	0 h	3 h	6 h	24 h
**11**	376.4 ± 21.00	342.8 ± 21.58	315.2 ± 21.66	276 ± 21.79
**12**	326.2 ± 25.32	300 ± 25.03	278.2 ± 25.76	245.2 ± 25.91
**13**	355 ± 24.59	322.8 ± 24.10	253.8 ± 23.45	231.4 ± 23.48
Pioglitazone	402.2 ± 28.7	363.4 ± 26.08	302.4 ± 26.87	232.2 ± 20.53
Metformin	441.8 ± 18.71	399.4 ± 17.72	289.4 ± 18.46	219.6 ± 18.40
Vehicle control	304.2 ± 36.81	308.2 ± 36.85	309 ± 37.92	310.4 ± 39.57
Diabetic control	322.2 ± 22.96	337 ± 23.59	347 ± 24.01	363.4 ± 24.0
Normal control	120.33 ± 7.76	125.66 ± 2.08	126.66 ± 3.05	129.33 ± 1.52

**Scheme 7:**
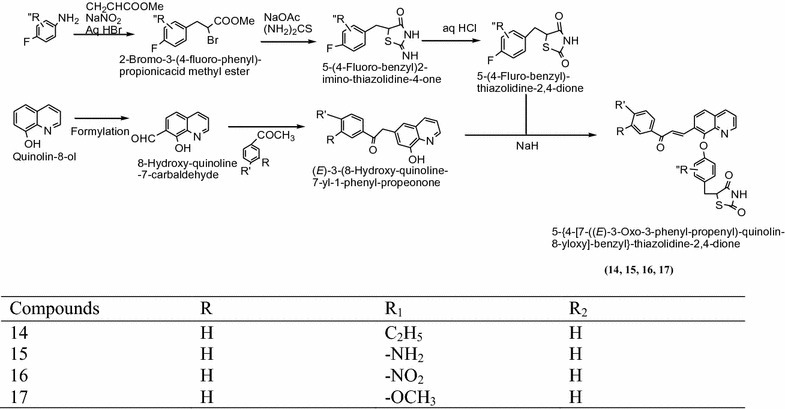
Synthesis of 5-{4-[7-((*E*)-3-Oxo-3-phenyl-propenyl)-quinolin-8-yloxy]-benzyl}-thiazolidine-2,4-dione

Srikanth et al. [11] synthesized an innovative sequence of thiazolidinediones using 4-fluoroaniline, methyl acrylate and thiourea using proper solvent as showed in Scheme 7. The In vitro antidiabetic activities of synthesized compounds were confirmed by tail vein method and ANOVA method. In this series compounds **14**, **15**, **16** and **17** showed significant activity as compared to standard drug rosiglitazone. The results of synthesized compounds presented in Table 7 (Srikanth et al. [11]).

**Table 7:** Antidiabetic activities of synthesized compounds (mg/dl)

Compounds	Blood glucose level (mean ± SE)
**14**	82.81 ± 1.115
**15**	86.31 ± 0.993
**16**	87.21 ± 1.233
**17**	97.91 ± 1.870
Rosiglitazone	65.58 ± 1.013

**Scheme 8:**
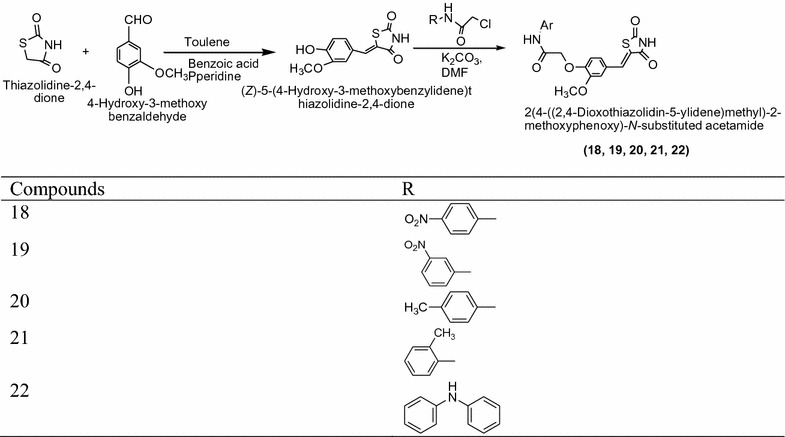
Synthesis of 2-(4-((2,4-Dioxothiazolidin-5-ylidene) methyl)-2-methoxyphenoxy)-*N*-substituted acetamide derivatives

Nikalje et al. [12] designed few thiazolidinediones derivatives from thiazolidindione via 4-hydroxy, 3-ethoxy benzaldehyde in ethanol, benzoic acid and piperidine using Scheme 8. The In vitro antidiabetic activity of synthesized compounds was confirmed by ANOVA, alloxan induced diabetic rat model and dunnet’ t test. From this series compounds **18**, **19**, **20**, **21**, and **22** showed better activity as compared to standard drug rosiglitazone. The results of synthesized compounds presented in Table 8 (Nikalje et al. [12]).

**Table 8:** Evaluation of hypoglycemic activity: effect of compound on % decrease in blood glucose in diabetic mice

Compounds	0 h	2 h	4 h	6 h	24 h
Control	252.53 ± 4.254	4.74 ± 0.68	7.9 ± 4.32	13.43 ± 2.68	3.18 ± 4.35
Piogiltazone	250.75 ± 5.21	31.07 ± 6.74	37.48 ± 5.37	45.41 ± 3.67	10.3 ± 6.53
**18**	252.79 ± 2.85	29.34 ± 4.53	36.52 ± 5.43	46.64 ± 4.52	6.70 ± 6.51
**19**	252.19 ± 4.35	24.7 ± 3.97	34.76 ± 6.51	37.89 ± 5.43	5.19 ± 7.74
**20**	254.38 ± 4.53	26.64 ± 5.28	34.26 ± 5.67	37.05 ± 4.62	4.19 ± 5.43
**21**	253.60 ± 5.64	22.9 ± 4.72	35.6 ± 5.53	40.41 ± 5.97	3.87 ± 6.53
**22**	252.73 ± 5.23	29.01 ± 6.54	36.47 ± 4.65	39.21 ± 5.74	3.0 ± 3.75

**Scheme 9:**
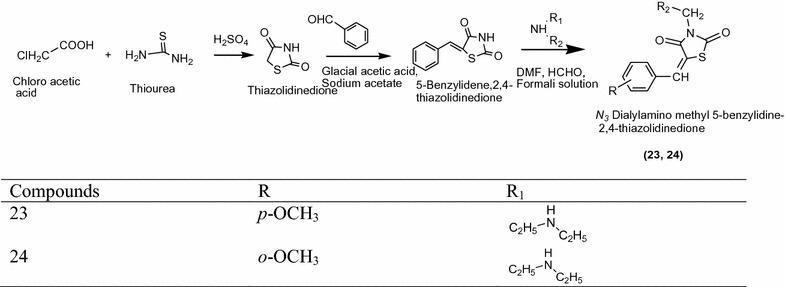
Synthesis of *N* ^3^-dialylamino methyl 5-benzylidine 2,4-thiazolidinedione derivatives

Jiwane et al. [13] synthesized a new series of thiazolidine-2,4-dione derivatives from 5-(benzylidene)thiazolidine-2,4-dione with *N* *N* ^*1*^-dimethylformamide in diethyl amino as presented in Scheme 9. The In vitro anitdiabetic activity of synthesized compound [3-((diethyl amino)methyl)-5-(4-methoxybenzylidine)thiazolidine-2,4-dione] were confirmed by alloxan induced diabetic rat model. From this series, compounds **23** and **24** showed remarkable activity as that of the standard rosiglitazine, which indicates that the substitution of α-amino methyl group at position-3 show different hypoglycemic activity. The results of most active derivatives showed in Table 9 (Jiwane et al. [13]).

**Table 9:** Hypoglycemic activity of synthesized derivatives

Compounds	Dose (mg/kg)	Mean blood glucose level (mg/dl)			% reduction in blood glucose level	
		Before 1st dose	After 2 h	After 4 h	After 2 h	After 4 h
**23**	50	400	56	48	86	88
**24**	50	275	63	79	72	65
Rosiglitazone	50	400	56	48	86	88

**Scheme 10:**
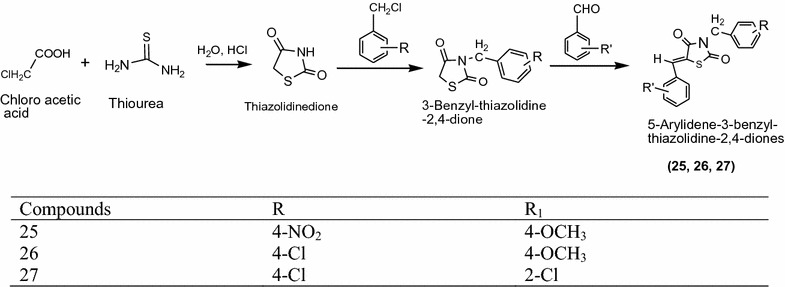
Synthesis of 5-Substituted-arylidene-3-substituted-benzyl-thiazolidine-2,4-dione derivatives

Grag et al. [14] designed novel thiazolidinediones derivative from 3-benzylthiazolidine-2,4-dione with selected various substituted aromatic aldehydes in ethanol, benzoic acid and piperidine using Scheme 10. In vitro antidiabetic activity of synthesized compound [5-arylidene-3-benzyl-thiazolidine-2,4-diones] was confirmed by ANOVA, alloxan induced diabetic rat model and dunnet’ t test. From this series compounds **25**, **26** and **27** showed highest activity because of methoxy group as compared to standard rosiglitazone. The results of synthesized compounds presented in Table 10 (Grag et al. [14]).

**Table 10:** Hypoglycemic activity of synthesized derivatives

Treatment (mg/kg)	Blood glucose level (mg/dl)			
	0 day	3rd day	5th day	7th day
**25**	86.11 ± 0.98	85.67 ± 0.58	84.68 ± 0.54	86.23 ± 0.48
**26**	188.23 ± 1.14	189.56 ± 0.98	185 ± 0.86	182.36 ± 1.25*
**27**	189.35 ± 1.18	206.38 ± 0.86	192.30 ± 1.2	188.36 ± 1.23
Rosiglitazone	194.99 ± 1.70	207.45 ± 0.69	189.64 ± 1.33	172.38 ± 2.24

* indicates high reduction in glucose level after seven days

**Scheme 11:**
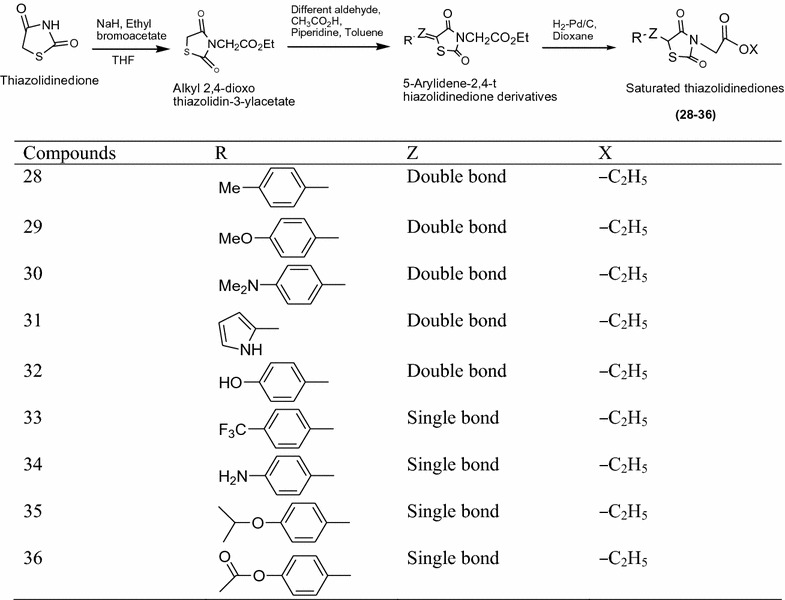
Synthesis of [5-(4-Methoxy-benzylidene)-2,4-dioxo-thiazolidin-3-yl]-acetic acid

Bhat et al. [15] synthesized a new series of thiazolidinediones derivatives derived from 5-arylidene-2,4-thiazolidinedione using Scheme 11. The In vitro antidiabetic activity of synthesized compound [5-(4-methoxy-benzylidene)-2,4-dioxo-thiazolidin-3-yl]-acetic acid] and [5-(substituted)-2,4-dioxo-thiazolidin-3-yl]-acetic acid substituted ester were performed by alloxan induced tail tipping method and SLM. Among them compounds **28**, **29**, **30**, **31**, **32**, **33**, **34**, **35** and **36** found to be most active or higher than rosiglitazone and metformin using as standard drug. The results of most active derivatives showed in Table 11 (Bhat et al. [15]).

**Table 11:** Antihyperglycemic activity profile of title compounds thiazolidine-2,4-dione derivatives

Compounds	Antihyperglycemic activity, SLM	PPARc	
		10 nmol	1000 nmol
**28**	− 22.1	9	9
**29**	− 22.2	7	8
**30**	− 15.8	–	–
**31**	+ 9.00	–	–
**32**	− 26.7	10	12
**33**	− 12.3	9	11
**34**	− 12.7	8	10
**35**	− 4.1	–	–
**36**	− 26.8	–	–
Rosiglitazone	11.6	92	248
Metformin	34.1	–	–

*PPAR*_*c*_ proxisome proliferator activated receptor

**Scheme 12:**
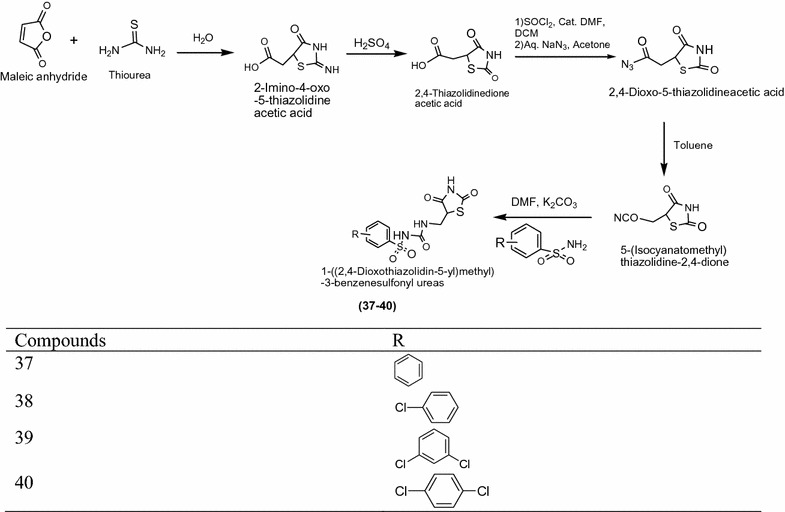
Synthesis of 1-((2,4-Dioxothiazolidin-5-yl)methyl)-3-substitued benzene sulphonyl ureas

Jawale et al. [16] synthesized innovative chain of thiazolidinediones derived from maleic anhydride and thiourea was treated with water using Scheme 12. The In vitro antidiabetic activity of synthesized compounds was performed by alloxan induced tail tipping method using wister rat, dunnet’ t test and SLM model. Among them compounds **37**, **38**, **39** and **40** found to be significant activity metformin using as standard drug. The results of most active derivatives showed in Table 12 (Jawale et al. [16]).

**Table 12:** Antidiabetic activity of synthesized compounds

Compounds	Dose (mg/dl)	% activity	Significance
**37**	100	15.8	p < 0.01
**38**	100	17.2	p < 0.01
**39**	100	14.3	p < 0.05
**40**	100	16.5	p < 0.01
Metformin	100	27.0	p < 0.001

### Thiazolidinedione derivatives as antimicrobial agents

Long-ago, contagious diseases caused by multidrug-resistant microorganisms have become a serious issue, representing a growing threat to human health and being a major problem in many countries worldwide. There has been a significant increase in clinical drug resistance over the past few decades, owing to exploitation of antimicrobial agents, thus many infectious disease can no longer be treated successfully with general anti-infective agents [17]. Modern therapies and management technique such as bone marrow or solid-organ transplants, and newer much aggressive chemotherapy have resulted in a rapidly inflating number of immune-suppressed patient. So, in order to meet above mentioned challenges, there is an urgent need for the development of novel antimicrobial agents [18].

**Scheme 13:**
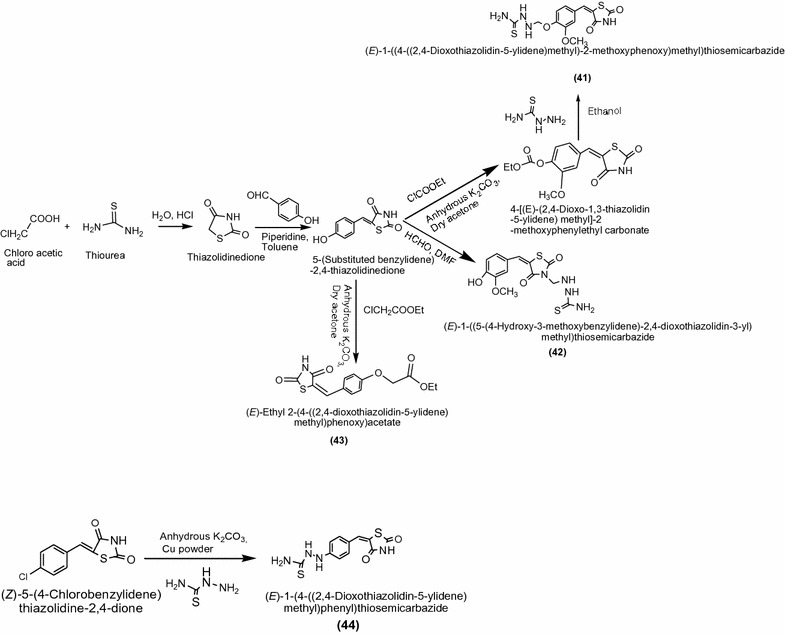
Synthesis of 5-Substituted benzylidenethiazolidine-2,4-dione

In this study, Nawale et al. [19] synthesized a new series of 5-Substituted 2,4-thiazolidinedione derivatives (Scheme 13) and evaluated for in vitro antimicrobial activity against two species of Gram-positive bacteria, *Bacillus subtilis*, *Staphylococcus aureus* and Gram-negative bacteria, *Pseudomonas aeruginosa* using broth dilution method. Among the synthesized derivatives, compounds **41**, **42**, **43** and **44** exhibited highest activity on all tested microorganisms. The results of synthesized compounds presented in Table 13 (Nawale et al. [19]).

**Table 13:** MIC (μg/ml) values for the screened thiazolidinediones compounds

Compounds	Microorganisms		
	*Bacillus subtilis*	*Staphylococcus aureus*	*Pseudomonas aeruginosa*
**41**	31.25	31.25	31.25
**42**	31.25	31.25	31.25
**43**	62.5	125	62.5
**44**	31.25	62.5	125
Streptomycin	3.90	3.90	3.90

**Scheme 14:**
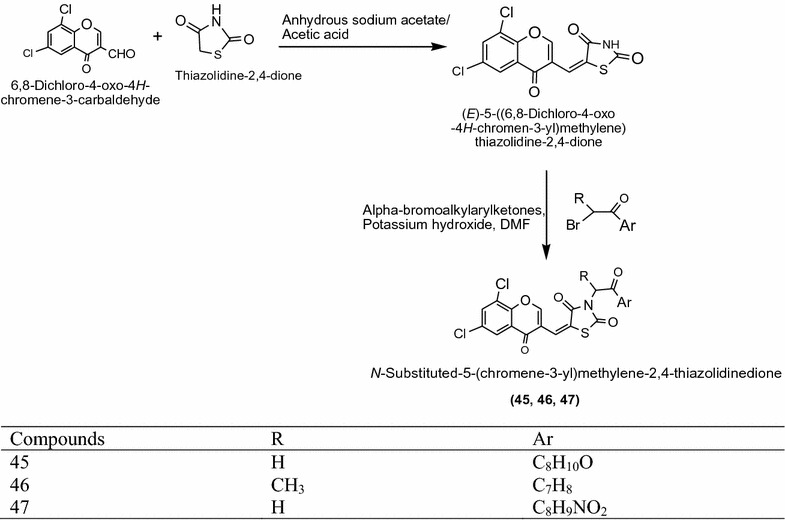
Synthesis of 5-(Chromene-3-yl)methylene-2,4-thiazolidinediones

Nastas et al. [20] synthesized a series of novel 5-(Chromene-3-yl)methylene-2,4-thiazolidinedione derivatives as presented in Scheme 14 and tested for its in vitro antimicrobial potency towards Gram-positive bacteria (*Listeria monocytogenes*, *Staphylococcus aureus)* and Gram-negative bacteria (*Escherichia coli*, *Salmonella typhi)* pathogenic bacteria and fungi (*Candida albicans*) using broth dilution method and the disk diffusion method. Among the synthesized derivatives, compounds **45**, **46** and **47** antimicrobial activity against all tested bacteria and fungi. The results of most active derivatives showed in Table 14 (Nastas et al. [20]).

**Table 14:** Antimicrobial activity of 5-(chromene-3-yl)methylene-2,4-thiazolidinediones

C_P_ 10/5/1(mg/ml)	Gram-positive		Gram-negative		Fungi
	*L. monocytogenes*	*S. aureus*	*E. coli*	*S. typhi*	*C. albicans*
**45**	18/22/18	22/12/12	12/14/14	15/19/20	20/18/18
**46**	22/22/20	24/28/28	18/18/16	20/18/16	18/18/16
**47**	28/28/28	28/28/28	18/18/18	18/18/18	22/22/22
Gentamicin	18	19	22	18	NT
Fluconazole	NT	NT	NT	NT	28

*NT* not tested

**Scheme 15:**
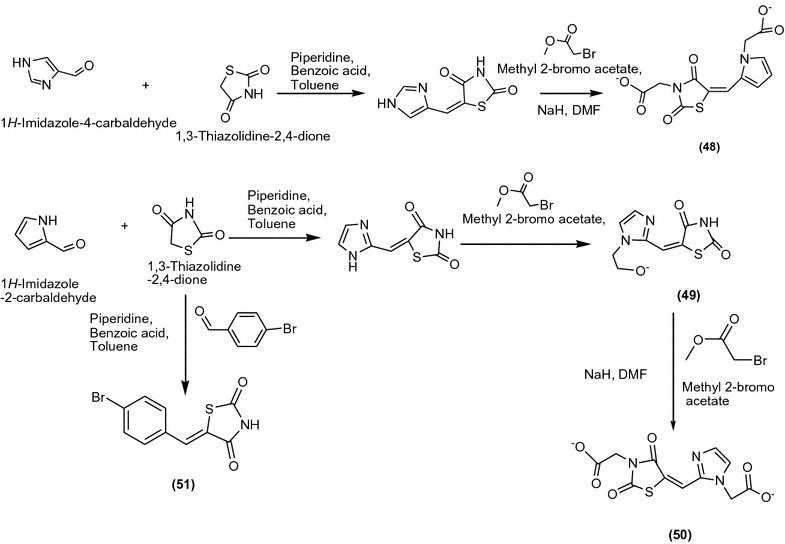
Synthesis of 5-(Substituted benzylidene)thiazolidine-2,4-dione and imidazolyl thiazolidinedione derivatives

Moorthy et al. [5] synthesized a series of novel imidazolyl thiazolidinedione derivatives (Scheme 15) and screened them for their in vitro antimicrobial activity towards Gram-positive (*S. aureus*, *S. epidermidis*, *M. luteus*, *B. cereus*) and Gram-negative (*E. coli*, *P. aeruginosa*, *K. pneumonia*) bacteria and fungi (*A.niger*, *A. fumigates)*. They were compared with standard drug ciprofloxacin and ketoconazole. Among the synthesized derivatives, compound **48** [Methyl-2-(4-((3-(2-methoxy-2-oxoethyl)-2,4-dioxothiazolidin-5-ylidene)methyl)1*H*-Imidazol-1-yl)acetate] showed potent activities towards *S*. *aureus*, *S*. *epidermidis*, *E*. *coli*, *P*. *aeruginosa*, *A*. *niger* and *A*, *fumigates* and **49** [Methyl-2-(2-((2,4-dioxothiazolidin-5-ylidene)methyl)-1*H*-imidazol-1-yl)acetate], **50** [Methyl-2-(2-((3-(2-methoxy-2-oxoethyl)2,4-dioxothiazolidin-5-yldiene)methyl)1*H*-imidazol-1-yl)acetate] and **51** [5-(4-Bromobenzylidene)thiazolidine-2,4-dione] showed good activity against all microorganism. The results of synthesized compounds presented in Table 15 (Moorthy et al. [5]).

**Table 15:** *In vitro* activity zone of inhibition (mm) of compounds

Compounds	Gram-positive bacteria		Gram-negative bacteria		Fungi	
	*S*. *aureus*	*S*. *epidermidis*	*E*. *coli*	*P*. *aeruginosa*	*A*. *niger*	*A*. *fumigates*
**48**	18 (1.9)	16 (1.4)	28 (1.6)	28 (0.56)	20 (8.8)	26 (2.3)
**49**	21 (22.1)	27 (22.2)	27 (21.5)	21 (21.5)	24 (20.7)	20 (22.6)
**50**	16 (2.7)	18 (3.39)	22 (9.2)	16 (1.4)	22 (8.2)	26 (3.4)
**51**	21 (22.1)	25 (22.2)	25 (21.5)	21 (21.5)	28 (21.6)	25 (21.7)
Ciprofloxacin	29 (0.2)	31 (0.39)	32 (0.2)	33 (0.25)	–	–
Ketoconazole	–	–	–	–	26 (6.1)	24 (0.23)

**Scheme 16:**
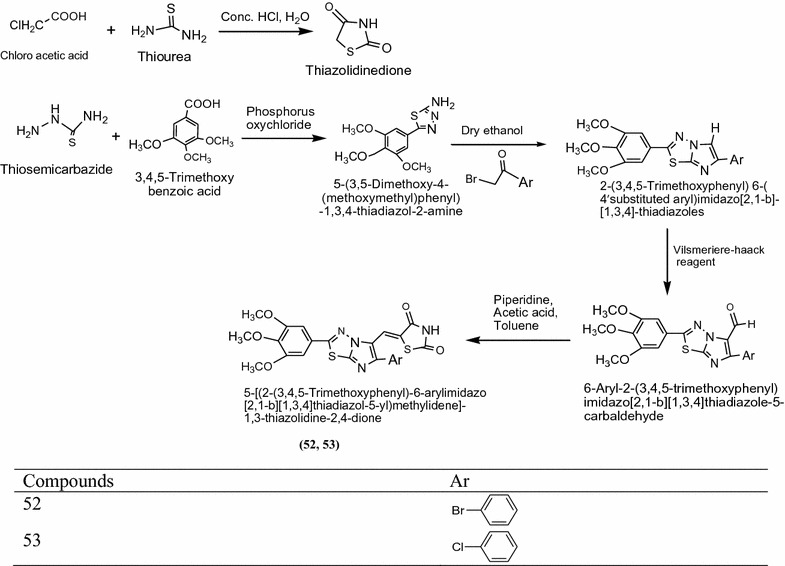
Synthesis of 5-[(2-(3,4,5-Trimethoxyphenyl)-6-arylimidazo[2,1-*b*][1,3,4]thiadiazol-5-yl)methylidene]-1,3-thiazolidine-2,4-dione

**Scheme 17:**
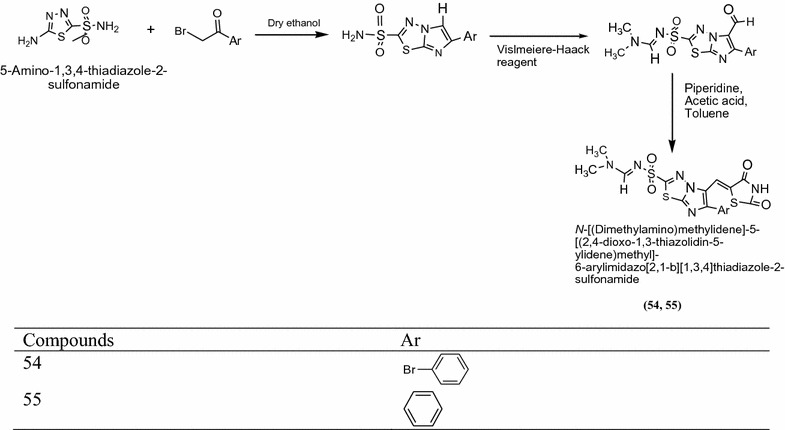
Synthesis of *N*-[(Dimethylamino)methylidene]-5-[(2,4-dioxo-1,3-thiazolidin-5-ylidene)methyl]-6-arylimidazo[2,1-*b*][1,3,4]thiadiazole-2-sulfonamide

Alagawadi et al. [21] designed some novel derivatives of imidazole fused with thiazolidine-2,4-dione and evaluated them for their antibacterial activity against Gram-positive bacteria *Staphylococcus aureus (S. a)*, *Enterococcus faecalis (E. f)* Gram-negative bacteria *Escherichia coli (E. c.) Pseudomonas aeruginosa (P. a.)* and antifungal activity *Candida albicans (C.a.) Cryptococcus neoformans (C. n.) Aspergillus flavus (A. f.)* and *Aspergillus niger (A. n.)* Among the screened compound the MIC value of compound **52** [5-{[2-(3,4,5-trimethoxyphenyl)-6-(4-bromophenyl)imidazo[2,1-*b*][1,3,4]thiadiazol-5-yl]methylidene}-1,3-thiazolidine-2,4-dione], **53** [5-{[2-(3,4,5-trimethoxyphenyl)-6-(4-chlorophenyl)midazo[1-*b*][1,3,4]thiadiazol-5-yl]methylidene}-1,3-thiazolidine-2,4-dione] (Scheme 16), **54** [*N*-[-(dimethylamino)methylidene]-5-[(2,4-dioxo-1,3-thiazolidin-5-ylidene)methyl]-6-phenylimidazo[2,1-*b*][1, 3, 4]thiazolie-2-sulfonamide] and **55** [*N*-[-(dimethylamino)methylidene]-5-[-(2,4-dioxo-1,3-thiazolidin-5-ylidene)methyl]-6-(4-bromophenyl)-imidazo[2,1-*b*][1,3,4]thiazole-2-sulfonamide] (Scheme 17) were showed potent activity against Gram-positive, Gram-negative bacterial strain and fungal strains. The significant results of these compounds are presented in Table 16 (Alagawadi et al. [21]).

**Table 16:** Antimicrobial activities of synthesized compounds

Compounds	Minimum inhibitory concentration (MIC) in μg/ml							
	*E*. *c*	*P*. *a*	*S*. *a*	*E*. *f*	*C*. *a*	*C*. *n*	*A. f*	*A*. *n*
**52**	256	256	32	32	4	8	4	4
**53**	128	64	32	32	4	8	32	32
**54**	128	32	8	4	1	2	4	4
**55**	64	64	8	8	4	8	4	4
Ampicillin	2	2	1	2	–	–	–	–
Ketoconazole	–	–	–	–	2	1	2	1

**Scheme 18:**
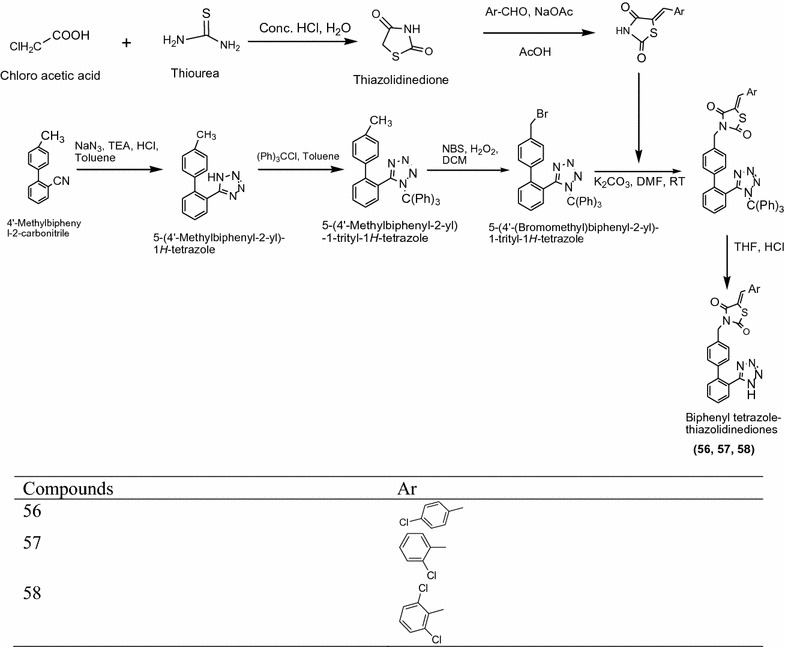
Synthesis of Biphenyl tetrazole-thiazolidinediones

Khan et al. [22] designed some novel biphenyl tetrazole thiazolidinedione derivatives (Scheme 18) and evaluated for their antimicrobial activity against bacterial strain (*Escherichia coli*, *Bacillus subtilis*). Antimicrobial activity result indicated that among the synthesized derivatives **56** [(*E*)-3-((20-(1*H*)-tetrazol-5-yl)biphenyl-4-yl)methyl)-5-(4-chlorobenzylidene)thiazolidine-2,4-dione], **57** ((*E*)-3-((20-(1*H*-tetrazol-5-y)biphenyl-4-yl)methyl)-5-(2-chlorophenylbenzylidene)thiazolidine-2,4-dione) and **58** [(*E*)-3-((20-(1*H*-tetrazol—5-yl)biphenyl-4-yl)methyl)-5-(2,6-dichlorobenzylidene) thiazolidine-2,4-dione] showed potent in vitro antimicrobial activity. The results of most active derivatives showed in Table 17 (Khan et al. [22]).

**Table 17:** Antibacterial activities of synthesized compounds

Compounds	MIC ± SLM (μg/ml)	
	*E*. *coli*	*B*. *subtilis*
**56**	20.75 ± 1.55	35.41 ± 2.41
**57**	19.41 ± 1.27	26.00 ± 1.96
**58**	8.58 ± 0.42	8.42 ± 0.51
Ciprofloxacin	25.00 ± 0.95	50.00 ± 1.75

**Scheme 19:**
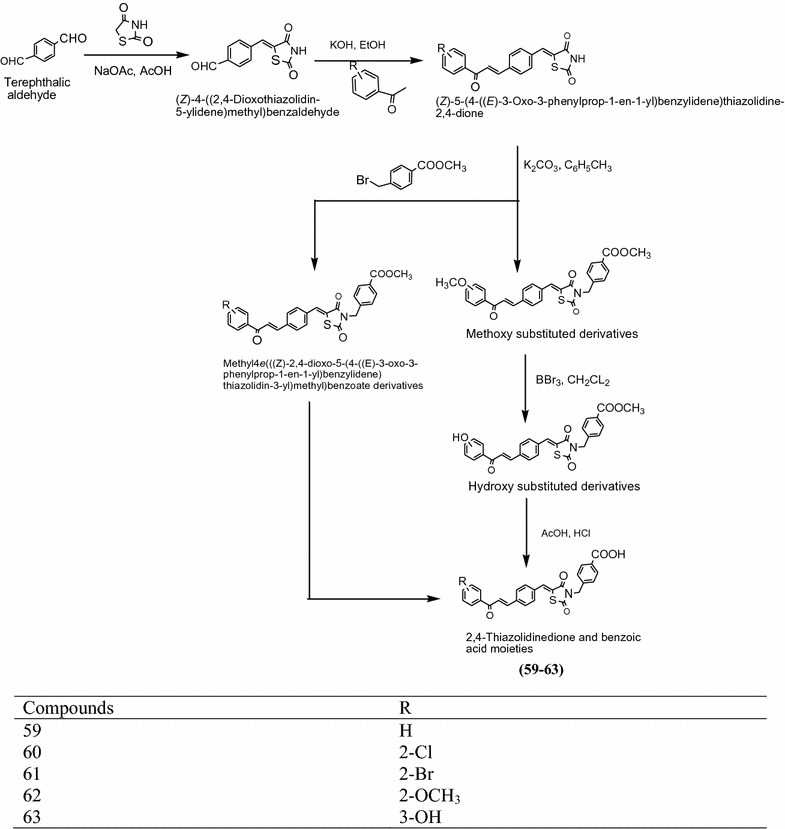
Synthesis of 4-(((*Z*)-5-((4-((*E*)-3-(Substituted)-3-oxoprop-1-en-1-yl)benzylidene)-2,4-dioxothiazolidin-3-yl)methyl)benzoic acid

Liu et al. [23] synthesized a series of new compound bearing 2,4-thiazolidinedione and benzoic moiety as presented in Scheme 19 and screened for their in vitro antimicrobial activity against bacterial strain (*Staphylococcus aureus* and *Escherichia coli*). Antimicrobial activity result indicated that among the synthesized derivatives, compounds **59**, **60**, **61**, **62** and **63** showed highest in vitro growth of inhibition against bacterial strains. The results of synthesized compounds presented in Table 18 (Liu et al. [23]).

**Table 18:** Inhibitory activities of novel compounds against bacteria

Compounds	*S*. *aureus*		*E*. *coli*	
	4220	530	1356	1682
**59**	1	2	> 64	> 64
**60**	1	2	> 64	> 64
**61**	2	4	> 64	> 64
**62**	2	4	> 64	> 64
**63**	2	4	> 64	> 64
Norfloxacin	2	2	16	16
Oxacillin	1	1	> 64	> 64

**Scheme 20:**
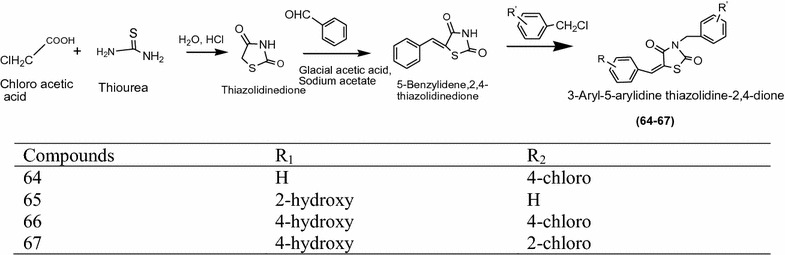
Synthesis of 3,5-Disubstituted thiazolidine-2,4-diones

Purohit et al. [24] synthesized a series of novel 3,5-disubstituted thiazolidinediones derivatives (Scheme 20) and evaluated its antibacterial activity against *Staphylococcus aureus*, *Enterococcus faecalis*, *Klebsiella pneumonia*, *Escherichia coli* and antifungal activity was performed against *Candia albicans*, *Aspergillus niger*, *Aspergillus flavus*. The screening results were compared with ciprofloxacin, norfloxacin for antibacterial and fluconazole, griseofulvin for antifungal activity respectively. Among the synthesized compounds **64**, **65**, **66** and **67** showed highest antimicrobial potency and their structure were. The significant results of these compounds are presented in Table 19 (Purohit et al. [24]).

**Table 19:** Antimicrobial activities of synthesized compounds

Compounds	Minimum inhibitory concentration (MIC μg/ml)						
	*S*. *aureus*	*E*. *faecalis*	*K*. *pneumonia*	*E*. *coli*	*C*. *albicans*	*A*. *niger*	*A*. *flavus*
**64**	4	4	250	500	16	16	8
**65**	4	31.25	62.5	62.5	31.5	1	8
**66**	2	4	> 500	> 500	4	8	8
**67**	1	1	62.5	62.5	4	4	2
Ciprofloxacin	2	2	1	2	–	–	–
Norfloxacin	10	3.1	0.1	10	–	–	–
Fluconazole	–	–	–	–	16	8	8
Griseofulvin	–	–	–	–	500	100	7.5

**Scheme 21:**
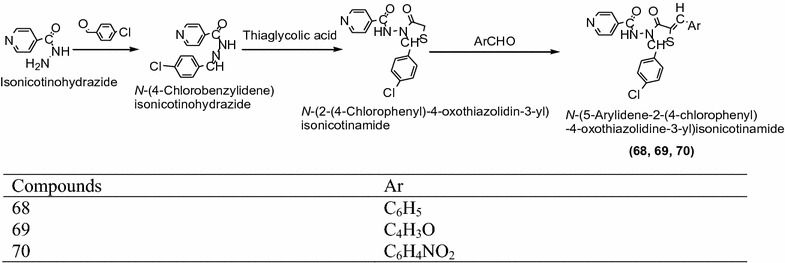
Synthesis of *N*-(5-Arylidene-2-(4-chlorophenyl)-4-oxothiazolidin-3-yl)isonicotinamide

Sharma et al. [25] synthesized a series of novel *N*-(-5-arylidene-2-(4-chlorophenyl)-4-oxothiazolidin-3-yl)isonicotnamide derivatives by knoevenagel condensation using Scheme 21 and assayed for antibacterial activity against *Escherichia coli*, *Staphylococcus aureus*, *Bacillus subtilis* and antifungal activity against *Candida albicans*, *Aspergillus niger*, *Saccharomyces cervesia* using turbidimetric method. Among the synthesized compounds **68** (*N*-(5-benzylidene-2-(4-chlorophenyl)-4-oxothiazolidin-3-yl)isonicotinamide), **69** (*N*-(2-(4-chlorophenyl)-5-(furan-2-ylmethylene)-4-oxothiazolidin-3-yl)isonicotinamide) and **70** (*N*-(5-(2-nitrobenzylidene)-2-(4-chlorophenyl)-4-oxothiazolidin-3-yl)isonicotinamide) result in wide spectrum antimicrobial activity against all the test bacteria and fungi using ciprofloxacin and clotrimazole as a standard drug respectively. The results of synthesized compounds presented in Table 20 (Sharma et al. [25]).

**Table 20:** Antimicrobial activities of synthesized compounds

Compounds	Minimum inhibitory concentration (MIC) in μg/ml					
	*E*. *coli*	*B*. *subtilis*	*S*. *aureus*	*A*. *niger*	*C*. *albicans*	*S*. *cerevisiae*
**68**	1.25	1.25	0.62	0.62	0.31	1.25
**69**	0.62	0.31	0.62	0.62	0.15	0.62
**70**	0.31	0.62	0.31	0.62	0.15	0.31
Ciprofloxacin	0.15	0.25	0.01	–	–	–
Clotrimazole	–	–	–	0.10	0.30	0.20

### Thiazolidine-2,4-dione derivatives as anti-inflammatory agents

The future of anti-inflammatory compound lies in the development of orally active drugs that decreases production or activities of pro-inflammatory cytokines. Anti-inflammatory compounds are normally used for curing of different infectious conditions. Therefore, the rate of incidence of disease limits its clinical use. Thus here is requirement of designing advance drugs with improved activity and long term relieve from chronic inflammatory condition [26]. The complete knowledge and understanding of the pivotal role of inflammation in seemingly untreated diseases has resulted in development of novel anti-inflammatory agents [27].

**Scheme 22:**
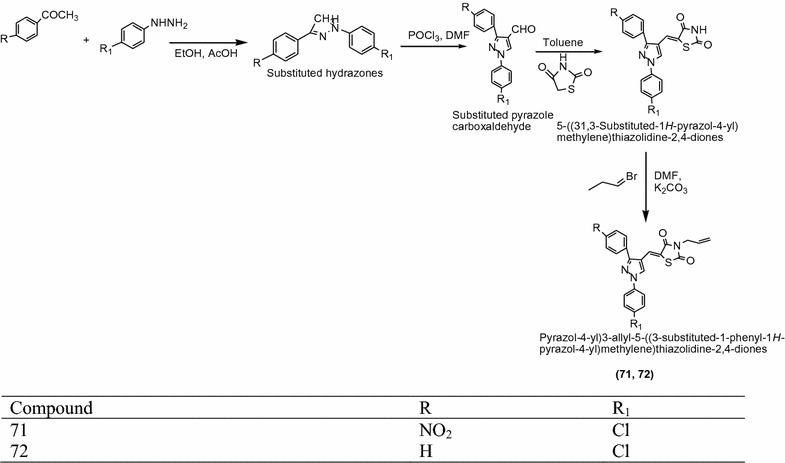
Synthesis of 3-Substituted benzyl-5-((3-substituted-1-phenyl-1*H*-pyrazol-4-yl)methylene)thiazolidine-2,4-diones

Youssef et al. [26] synthesized some novel active pyrazolyl-2,4-thiazolidinedione derivatives (Scheme 22) followed by their in vitro anti-inflammatory evaluation. Among them, compounds **71** and **72** [(*Z*)-3-allyl-5-((3-(4-chlorophenyl)-1-phenyl-1*H*-pyrazol-4-yl(methylene)thiazolidine-2,4-dione] showed moderate to good anti-inflammatory activity using celecoxib as standard and turpentine oil as control. The results of potent derivatives presented in Tables 21, 22 and 23 (Youssef et al. [26]).

**Table 21:** Cyclooxygenase inhibition activity of synthesized compound

Compounds	Concentration (Um) (no. of experiments)	COX-1 activity (% inhibition)	COX-2 activity (% inhibition)
**71**	10 (3)	28.4 ± 11.6	19.4 ± 8.2
**72**	10 (3)	26.5 ± 6	13.6 ± 1.1
Celecoxib	10 (3)	0.3 ± 2.5	30.8 ± 5.9

**Table 22:** Inflammation reduction results of synthesized compounds in Formalin induced rat paw edema bioassay

Compounds	Volume of edema (ml)				
	0 h	1 h	2 h	3 h	4 h
**71**	0.31 ± 0.001	0.44 ± 0.01 (24)	0.44 ± 0.01 (46)	0.46 ± 0.003 (68)	0.46 ± 0.02 (68)
**72**	0.33 ± 0.02	0.41 ± 0.01 (53)	0.42 ± 0.01 (63)	0.46 ± 0.01 (72)	0.49 ± 0.01 (66)
Control	031 ± 0.01	0.40 ± 0.01	0.55 ± 0.01	0.78 ± 0.01	0.78 ± 0.008
Celecoxib	0.31 ± 0.01	0.41 ± 0.005 (41)	0.43 ± 0.02 (50)	0.50 ± 0.005 (60)	0.48 ± 0.03 (68)

**Table 23:** Inflammation reduction results of synthesized compounds in turpentine oil induced granuloma pouch bioassay in rat

Compounds	Volume of exudates (ml)	% inhibition
**71**	1.12 ± 0.06	51
**72**	1.12 ± 0.06	50
Control	2.28 ± 0.07	–
Celecoxib	1.05 ± 0.10	54

**Scheme 23:**
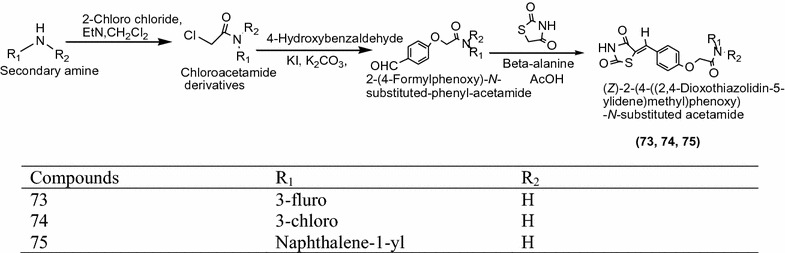
Synthesis of (*Z*)-2-(4-((2,4-Dioxothiazolidin-5-ylidene)methyl)phenoxy)-*N*-substituted acetamide

Ma et al. [28] synthesized a series of novel 5-benzylidene thiazolidine-2,4-dione derivatives as presented in Scheme 23 and screened for in vitro inflammation reduction activity. Among the synthesized derivatives, compounds **73** [(*Z*)-2-(4-((2,4-dioxothiazolidin-5-ylidene)methyl)phenoxy)-*N*-(3-fluorophenyl)acetamide], **74** [(*Z*)-*N*-(3-chlorophenyl)-2-(4-((2,4-dioxothiazolidin-5-ylidene)methyl)phenoxy)acetamide] and **75** [(*Z*)-2-(4-((2,4-dioxothiazolidin-5-ylidene)methyl)phenoxy)-*N*-(naphthalene-1-yl)acetamide] were found to be most active anti-inflammatory agent compared to indomethacin as the standard. The results of potent compounds are accessible in Table 24 (Ma et al. [28]).

**Table 24:** Anti-inflammatory activities of synthesized derivatives

Compounds	No inhibition (%) ± SD
**73**	41.5 ± 3.1
**74**	80.9 ± 5.0
**75**	70.9 ± 13.6
Indomethacin	63.2 ± 4.0

### Thiazolidinedione derivatives as anticancer agents

Cancer is a genetic disorder that has always been a major threat all over the world and has been characterized by proliferation of abnormal cells and exhibiting an increasing mortality rate globally and being characterized by rapid formation of abnormal cells and spreading through metastasis to different organs [29, 30]. Currently available treatment (chemotherapy and radiotherapy) for most types of cancer only provide temporary therapeutic benefits as well as being limited by a narrow therapeutic index, remarkable toxicity and acquired resistance [31]. In recent times, advance in clinical researches for anticancer agents have been increased and as neoplastic cells are the anomalous proliferation of cells in the body which cause cancer, various effective compounds derived from natural products have been isolated and developed as anticancer agents. These chemical compounds are formulated with a view to create effective action with minimum side effects against cancer [32].

**Scheme 24:**
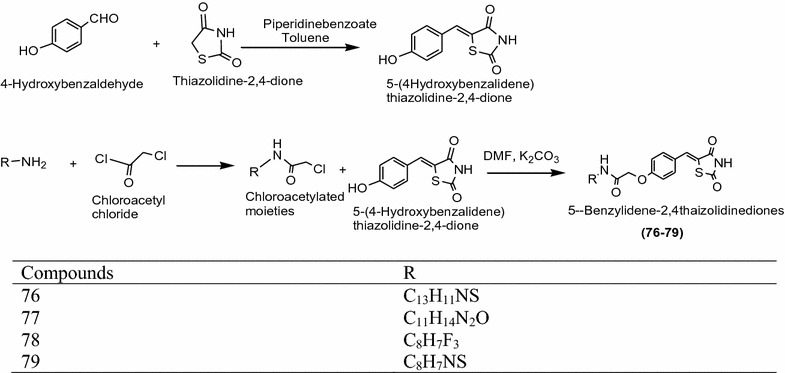
Synthesis of 5-Benzylidene-2,4-thiazolidinedione derivatives

Patil et al. [33] developed a novel class of 5-benzylidene-2,4-thiazolidinediones using Scheme 24. The synthesized derivatives were screened for the anticancer activity against K-562 (human leukemia), MCF-7 (human breast cancer), HepG-2 (human hepatoma), PC-3 (human prostate cancer), GURAV (human oral cancer) and KB (human nasopharyngeal cancer) cell lines by SRB protein assay. Among this series, **76**, **77**, **78** and **79** displayed the most potent anticancer activity compared with doxorubicin. The results of synthesized compounds presented in Table 25 (Patil et al. [33]).

**Table 25:** Anti-tumor activities of synthesized derivatives in different cell lines

Compounds	Diseases	Cancer cell line	Log GI_50_ (μM)	Log_10_ TGI (μM)
**76**	Leukemia	K-562	> − 0.4	> − 4.0
	Breast cancer	MCF-7	− 4.53	> 4.0
	Hepatoma	HEPG-2	> − 4.0	> 4.0
	NSC lung cancer	HOP-62	− 6.72	− 4.54
	Prostate cancer	PC-3	− 4.53	> − 4.0
	Oral cancer	GURAV	> − 4.0	> − 4.0
	Nasopharyngeal cancer	KB	> − 4.0	> − 4.0
**77**	Leukemia	K-562	> − 4.0	> − 4.0
	Breast cancer	MCF-7	> − 4.0	> − 4.0
	Hepatoma	HEPG-2	> − 4.0	> − 4.0
	NSC lung cancer	HOP-62	− 6.73	> − 4.0
	Prostate cancer	PC-3	> − 4.0	> − 4.0
	Oral cancer	GURAV	> − 4.0	> − 4.0
	Nasopharyngeal cancer	KB	> − 4.0	> − 4.0
**78**	Leukemia	K-562	− 6.72	> − 4.0
	Breast cancer	MCF-7	− 6.71	− 4.52
	Hepatoma	HEPG-2	> − 4.0	> − 4.0
	NSC lung cancer	HOP-62	> − 4.0	> − 4.0
	Prostate cancer	PC-3	− 5.60	> − 4.0
	Oral cancer	GURAV	− 6.73	− 4.52
	Nasopharyngeal cancer	KB	− 5.65	> − 4.0
**79**	Leukemia	K-52	> − 4.0	> − 4.0
	Breast cancer	MCF-7-5	− 4.60	> − 4.0
	Hepatoma	HEPG-2	> − 4.0	> − 4.0
	NSC lung cancer	HOP-62	− 6.77	> − 4.0
	Prostate cancer	PC-3	− 4.55	− 4.54
	Oral cancer	GURAV	> − 4.0	> − 4.0
	Nasopharyngeal cancer	KB	> − 4.0	> − 4.0
Doxorubicin	Leukemia	K-562	− 5.59	> − 4.0
	Breast cancer	MCF-7	− 6.88	− 5.68
	Hepatoma	HEPG-2	> − 7.0	− 6.87
	NSC lung cancer	HOP-62	− 6.91	− 4.45
	Prostate cancer	PC-3	− 6.96	− 5.68
	Oral cancer	GURAV	− 6.97	− 6.80
	Nasopharyngeal cancer	KB	> − 7.0	− 6.85

**Scheme 25:**
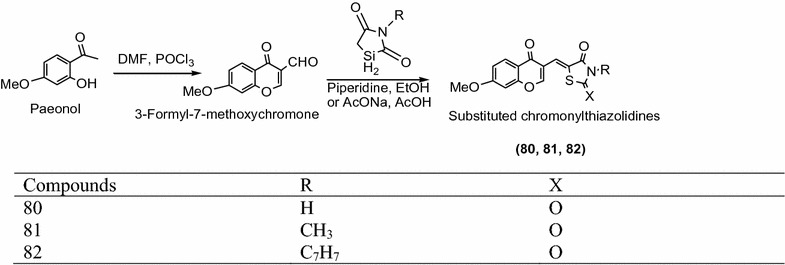
Synthesis of 5-((7-Methoxy-4-oxo-4*H*-chromen-3-yl)methylene) substituted thiazolidine-2,4-dione

Anh et al. [34] designed a chain of novel chromony thiazolidinediones derived from knoevenagel condensation reaction between 3-formyl-7-methoxy chromone with different thiazolidinedione derivatives as presented in Scheme 25. These synthesized derivatives were screened for their cytotoxic activity against Hep-G_2_ (heptocellular carcinoma), HC-60 (acute promyeloid carcinoma), KB (epidermoid carcinoma), LLC (lewis lung carcinoma), LNCaP (hormone dependent prostate carcinoma), MCF-7 (breast cancer), SW-480 (colon adenocarcinoma) cell lines using the MTT [3-(4,5-dimethylthiazol-2-yl)-2,5-diphenyl-2*H*-tetrazolium bromide] assay. In this series compounds **80**, **81** and **82** showed highest cytotoxic activity against cancer cell lines. The results of potent compounds are presented in Table 26 (Anh et al. [34]).

**Table 26:** Cytotoxicity of synthesized thiazolidinediones

Compounds	IC_50_ (μg/ml)							
	HepG_2_	HC-60	KB	LLC	LNCaP	LU-1	MCF-7	SW-480
**80**	> 100	82.2 ± 4.5	44.1 ± 3.6	87.4 ± 6.3	77.4 ± 5.8	52.9 ± 3.4	66.0 ± 2.7	71.4 ± 3.6
**81**	86.3 ± 6.4	75.3 ± 3.9	84.6 ± 4.2	> 100	81.6 ± 6.3	> 100	32.8 ± 1.4	90.1 ± 4.8
**82**	78.4 ± 5.8	92.3 ± 5.3	74.1 ± 5.1	90.1 ± 7.7	84.2 ± 4.1	65.5 ± 4.1	52.7 ± 3.6	85.4 ± 7.4
Ellipticine	1.45 ± 0.08	0.56 ± 0.04	0.43 ± 0.05	0.98 ± 0.04	0.86 ± 0.06	1.29 ± 0.11	0.49 ± 0.04	0.64 ± 0.05

**Scheme 26:**
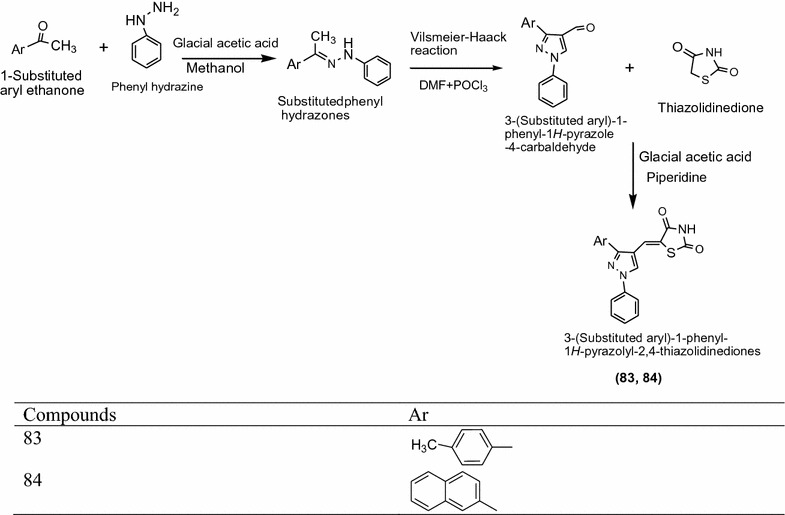
Synthesis of 3-(Substituted aryl)-1-phenyl-1*H*-pyrazolyl-2, 4-thiazolidinediones

Kumar et al. [35] synthesized a series of novel 3-(substituted aryl)-1-phenyl-1*H*-pyrazolyl-2,4-thiazolidinedione derivatives using Scheme 26. These synthesized derivatives were screened for their cytotoxic activity against lung and breast cancer cell lines using standard doxil. In this series **83** and **84** showed highest cytotoxic activity against cancer cell lines. The results of potent compounds are presented in Table 27 (Kumar et al. [35]).

**Table 27:** IC_50_ value of synthesized derivatives against cancer cell lines

Compounds	IC_50_ (μM)		
	A549	MCF-7	DU145
**83**	05.12	09.16	43.17
**84**	06.83	4.44	59.29
Doxil	07.92	08.12	07.22

### Thiazolidinedione derivatives as antioxidant agent

Free radicals produced in several biochemical reactions, cellular metabolism are negotiator for several infections and diseases like atherosclerosis, tumor as well as heart disease. Free radicals are not only formed by normal cellular processes but also produced by exposure of numerous chemical substances (polycyclic aromatic hydrocarbon, cadmium, lead, etc.), radiations, cigarette, smoke, and higher obese food. Usually free radical development is stopped by beneficial compounds known as antioxidant. Antioxidants deactivate free radicals before they attack the cell. Natural antioxidants are body detoxifiers and natural cleansers. They convert toxins of body to harmless waste products. They protect body from many diseases like cancer, heart attack and absorb bad cholesterol. Synthetic antioxidants such as BHT (butylated hydroxytoluene) and BHA (butylated hydroxyanisole), are effective as a antioxidants are also present and are used in several industries but there use has been limited because they can cause cancer as well as other side effects. So there use is decreased in food, cosmetic and pharmaceutical products. Thus, in present there is need for the oxidation inhibitor compounds [18, 36, 37].

**Scheme 27:**
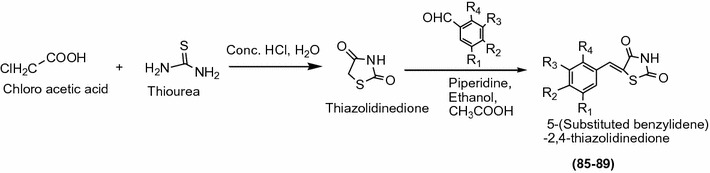
Synthesis of 5-Benzylidene 2,4-thiazolidinediones

Hossain et al. [37] synthesized a series of novel *O*-prenylated and *O*-geranylated derivatives of 5-benzylidene2,4-thiazolidinedione by knoevengeal condensation as showed in Scheme 27 and evaluated for their antioxidant activity. Among the synthesized derivatives, compounds **85**, **86**, **87**, **88** and **89** were found to be most active antioxidant agent. The significant results of potent compounds are given in Table 28 (Hossain et al. [37]).

**Table 28:** Inhibition of DPPH radical by synthesized compounds

Compounds	R_1_	R_2_	R_3_	R_4_	IC_50_ (μM)
α-Tocopherol	H	Hydroxyl	H	H	2.3
**85**	Methoxy	Hydroxyl	H	H	2.49
**86**	Methoxy	Hydroxyl	Methoxy	H	2.85
**87**	Methoxy	PRO	H	H	17.89
**88**	Methoxy	PRO	Methoxy	H	4.08
**89**	H	GRO	H	H	9.8

*DPPH* 1,1-diphenyl-2-picrylhydrazyl

**Scheme 28:**
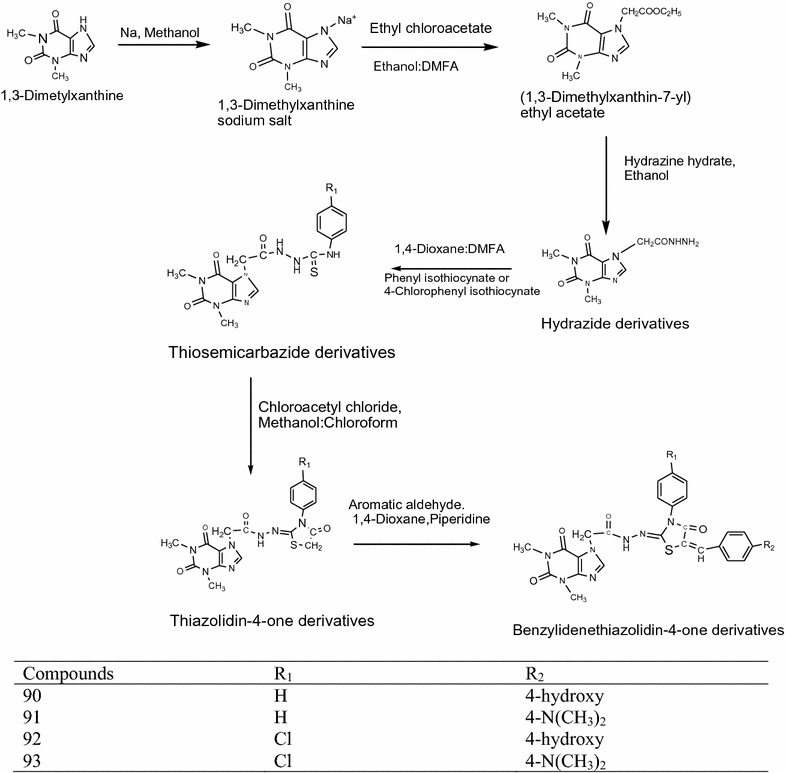
Synthesis of 2-{2-[2-(1,3-Dimethylxanthin-7-yl)acetyl]hydrazono}-3-(4-R1-phenyl-5-(R2-benzyliden)thiazolidin-4-ones

Lupascu et al. [4] designed a chain of novel thiazolidinediones containing xanthine moiety (Scheme 28) and evaluated for antioxidant potential using in vitro models such as DPPH radical scavenging assay and ABTS [2,2-azino-bis-(3-ethyl benzothiazoline-6-sulfonic acid] radical scavenging assay method. Among the synthesized derivatives **90**, **91**, **92** and **93** showed highest antioxidant activity. The results of potent derivatives are given in Table 29 (Lupascu et al. [4]).

**Table 29:** Antioxidant activities of the synthesized derivatives

Compounds	EC_50_ mg/ml
**90**	0.025 ± 0.0012
**91**	0.022 ± 0.0013
**92**	0.033 ± 0.0014
**93**	0.026 ± 0.0028
Ascorbic acid	0.0067 ± 0.0003

### Thiazolidinedione derivatives as anti-tubercular agents

In present day, treatment of tuberculosis diseases (TB) is chief and challenging problem because of resistance to present regimen and also appearance of drug-resistance strains in tuberculosis like *mycobacterium tuberculosis*, is transmitted by air and can affected all organ of the body, especially the lungs [38]. The association of tuberculosis with HIV infection is so dramatic that in some cases, nearly two-third of the patients diagnosed with the tuberculosis is also HIV-1 seropositive [39]. The current drug therapy for TB is long and complex, involving multidrug combinations (usually isoniazid, rifampin, ethambutol, and pyrazinamide for the initial 2 months and rifampin and isoniazid for an additional 4 months) [40]. There is also an alarming increase in cases of TB caused by multidrug-resistant strains of *M*. *tuberculosis*. Thus, there is a need for new drugs targeting enzymes essential to mycobacterium survival [41, 42].

**Scheme 29:**
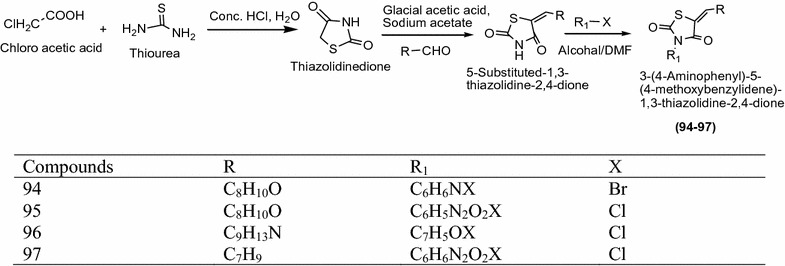
Synthesis of 3,5-Disubstituted-1,3-thiazolidine-2,4-dione

Chilamakuru et al. [42] synthesized a series of novel 3,5-disubstituted-2,4-thiazolidinediones as presented in Scheme 29 and appraised for anti-tubercular activities with pyrazinamide and streptomycin as the standard drug. Among all the synthesized derivatives, compounds **94**, **95** [3-(2-amino-5-nitrophenyl)-5-(4-methoxybenzylidene)-1,3-thiazolidine-2,4-dione], **96** [3-tert-butyl-5-(4-methoxybenzylidene)-1,3-thiazolidine-2,4-dione] and **97** showed the maximum antitubercular activity against *Mycobacterium tuberculosis* H37Rv strain. The results of synthesized compounds presented in Table 30 (Chliamakuru et al. [42]).

**Table 30:** Anti-tubercular activity of synthesized derivatives

Compounds	MIC μg/ml
**94**	12.5
**95**	12.5
**96**	12.5
**97**	12.5
Pyrazinamide	3.125
Streptomycin	6.25

**Scheme 30:**
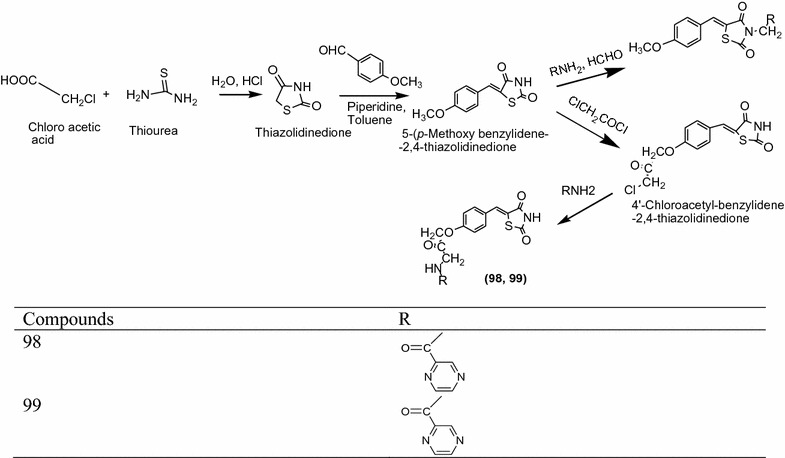
Synthesis of 4-Substitutedacetyl-benzylidene-2,4-thiazolidinediones

Pattan et al. [43] integrating a series of novel substituted thiazolidinediones via knoevenageal condensation reaction as presented in Scheme 30 and evaluated for their antitubercular activites by middle book 7H9 agar medium assay with streptomycin as the standard drug. Among all the synthesized derivatives, compounds **98** [(*Z*)-*N*-(3-(4-((2,4-dioxothiazolidin-5-ylidene)methyl)phenoxy)-2-oxopropyl)pyrazin-2-carboxamide] and **99** [(*Z*)-5-(4-methoxybenzylidene)-3-(2-oxo-2-(pyrazin-2-yl)ethyl)thiazolidine-2,4-dione] showed the maximum antitubercular activity against *Mycobacterium tuberculosis* H37Rv strain. The results of synthesized compounds presented in Table 31 (Pattan et al. [43]).

**Table 31:** Antitubercular activity of synthesized derivatives

Compounds	25 μg/ml	50 μg/ml	100 μg/ml
**98**	Resistant	Resistant	Sensitive
**99**	Resistance	Resistance	Sensitive
Streptomycin	Sensitive	Sensitive	Sensitive

## Conclusion

Appraisal of literature reports reveals that thiazolidinediones and its derivatives represent an important class of compound in the medicinal field with various therapeutic potentials, i.e., antidiabetic, antimicrobial, anti-inflammatory, anticancer, antioxidant and antitubercular, antiviral, anti-malarial, anti-HIV and anti-convulsant activities etc. which created immense interest among researchers to synthesized variety of thiazolidinediones. This review focuses especially on synthesized active compounds of thiazolidinediones having different pharmacological activities playing an important role in the medicinal field. These most active thiazolidinediones derivatives may be taken as leads to discover novel agents with therapeutic potential in the future.
